# Funding system reform for excellence in science: an interview with Jinghai Li, President of NSFC

**DOI:** 10.1093/nsr/nwy165

**Published:** 2019-01-31

**Authors:** Zhonghe Zhou, Weijie Zhao

**Affiliations:** 1 Director of the Institute of Vertebrate Paleontology and Paleoanthropology, Chinese Academy of Sciences and Associate Editor-in-Chief of NSR; 2 NSR news editor based in Beijing

## Abstract

The National Natural Science Foundation of China (NSFC) is the major funding agency for China's basic research in natural science. The total budget for NSFC was 26.7 billion Yuan (RMB) in 2017, accounting for 27% of China's total investment in basic research. In the past decades, continuous increases in the National Natural Science Fund and other funding programs provided strong support for the rapid growth in China's science and technology (S&T). In the second half of 2018, NSFC unveiled a deep reform plan that aims to build a fair, efficient and standardized new funding system that meets the demands of excellence in science in the twenty-first century in 5–10 years. Why did NSFC propose this reform? What are the major tasks of this reform? And how would NSFC implement this reform? All-in-all, this reform would not only have profound effect on S&T in China but also matters the world for the global collaborative efforts for the science. Recently, *National Science Review* had an exclusive interview with Jinghai Li, President of NSFC and Academician of the Chinese Academy of Sciences, to learn his views and perspectives of the future of NSFC.

## REFORM: THE BACKGROUND AND THREE MAJOR TASKS

**NSR:** What is the background of this reform? What are the issues this reform aims to deal with?

**Li:** The NSFC has been operating for 32 years since its establishment in 1986. The overall development of China's S&T and the funding portfolio of NSFC have undergone great changes ever since then. Firstly, NSFC has new missions in the new era. Secondly, the annual budget of NSFC grows from 80 million Yuan to 28 billion Yuan, representing a growth of several hundred times. Thirdly, the research paradigm of global science, including methods, contents and scopes of research, has also changed radically. In addition, international collaboration is gaining momentum and scientific issues dealing with global challenges have become a focal point of scientific research.

To adapt to these new challenges and new needs, we believe that it is high time to carry out a thorough reform of the current funding system. Otherwise we will miss the opportunity, and even slow the pace to build China into a strong power in S&T.

After considerable studies and discussions, we identified three major reform tasks: identifying funding categories, improving evaluation mechanisms and optimizing the layout of research areas for our funding system.

The first task is to identify funding categories. As a funding agency, NSFC must clearly identify its funding priorities and categories so as to play a guiding role for the research and behaviors of applicants. Clear identification of funding categories will also help to improve the quality of applications and make the evaluation process more efficient and fairer.

The second task is to set up a category-specific, accurate, fair and effective evaluation mechanism, which guarantees timely support to original ideas of the best scientific merits.

**Figure 1:**
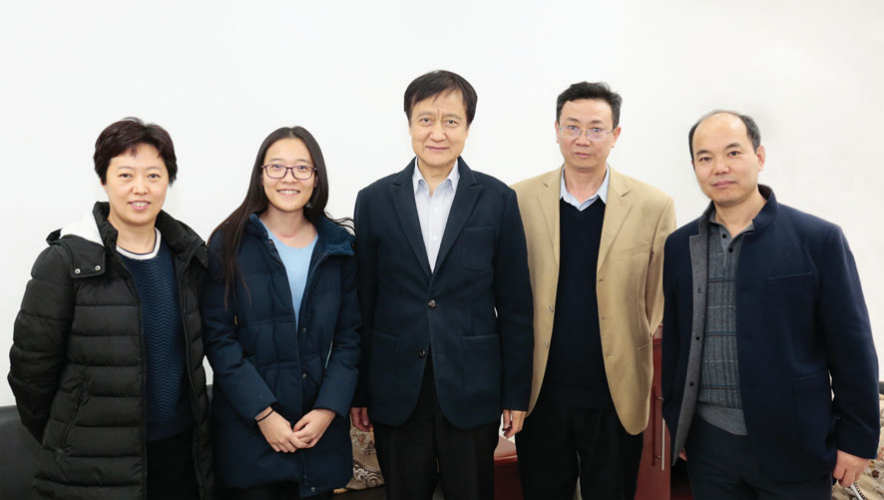
Jinghai Li (center) with the interviewers and NSR staff *(Photo: Tianxiao Gao)*.

The third, probably the most challenging task is to optimize the layout of research areas. Our current disciplinary codes for application are extremely complex. We have large numbers of codes referring to areas of diverse sizes. The codes treat research areas as separated domains without reflecting their complicated inter-connections. This brings considerable difficulties to the application and evaluation process. By optimizing this system, we will make our research coding system clearer and more in accordance with the inherent logic and landscape of the knowledge system. It will not only promote interdisciplinary research and avoid knowledge gap between disciplines, but also provide an important reference to the educational system and research institutes in China.

Besides these three tasks, we are also planning to rearrange NSFC funding programs, including our talent programs and the Basic Science Center Program. In recent years, there have been some controversies over NSFC's talent programs, especially the National Science Fund for Distinguished Young Scholars (DYS). How to respond to the concerns of the scientific community and give better play to the talent programs, and how to encourage multi-source investment for S&T are questions that we need to take into consideration.

Through this reform, we hope to build a conceptually advanced, professionally managed, fair and efficient funding system which meets the demands of the new paradigm of science.

## IDENTIFY FUNDING CATEGORIES

**NSR:** The reform plan identified four major funding categories: creative and timely ideas, research focusing on frontiers of science in unique ways, application-driven basic research and transdisciplinary leading-edge research. How is the funding proportion of these four areas allocated?

**Li:** The four categories are classified based on the attributes of scientific problems, but they are not mutually exclusive. A scientific problem can be a second-category frontier problem and a third-category application-driven problem at the same time. The 2015 Nobel Prize winner invention, the blue light-emitting diode (LED), is a good example: it started from frontier scientific research and reached the application end.

We may gradually find a roughly appropriate proportion based on the application and evaluation of the next few years. My personal estimation is that the application number and funding for application-driven problems may increase. This prediction comes from previous experience as well as NSFC's emphasis on application-driven research. For example, we have already collaborated with companies and local governments to set up a number of joint funds, mostly dedicated to application-driven research.

**NSR:** According to the official data, China's investment for basic research is relatively low compared to developed countries, accounting for only 5.2% of the total Research and Development (R&D) investment. The reform of NSFC aims to further support application-driven research. Will this weaken the support to basic research in China?

**Li:** Indeed, the support to basic research in China is still insufficient. The ratio of funding to basic research in the total R&D investment is low, and this is due to many factors. China's investment in basic research basically comes from the national finance. We don't have a mechanism to guarantee multi-source investment in S&T. For example, except for several major companies, few Chinese companies pay attention to basic research. Huge amount of investment of companies goes to application-oriented research and development.

On the other hand, we should keep in mind that there is no clear boundary between basic research and applied research.

There are always basic scientific questions to be solved behind every single application problem. NSFC concentrates on basic research. As long as the research deals with scientific problems, it should be considered as basic research. Thus, there is no real contradiction between emphasis on application-oriented scientific problems and support to basic research. The emphasis on application-driven research will not weaken the support to basic research in China.

**NSR:** China is facing many technological bottlenecks. How could NSFC, dedicated to supporting basic research, help to break the bottlenecks?

**Li:** As I have just mentioned, NSFC is going to strengthen its support to the application-driven scientific problems, which exactly targets at the bottlenecks. For the bottlenecks, an important issue is that we usually know ‘what is the technology gap we need to solve' but we cannot tell ‘what is the basic scientific problem that need to be solved beforehand'. That is why we cannot overcome the bottlenecks. NSFC can play a better role through mobilizing the scientific community to identify the basic scientific problems behind every technological need. In this aspect, I feel NSFC still has a long way to go.

## IMPROVE EVALUATION MECHANISMS

**NSR:** The reform proposed a ‘Responsibility + Credit + Contribution (RCC)' peer-review system. How will such a system be built?

**Figure 2:**
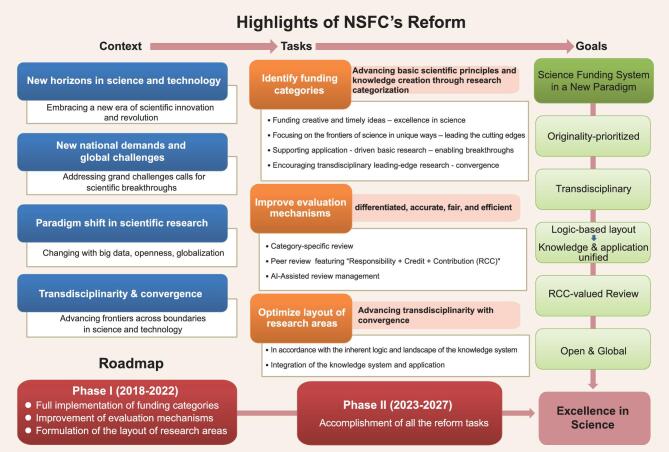
Highlights of NSFC's reform.

**Li:** The readjustment of the existing evaluation system can be a very complicated process, but it is something we must do. In NSFC, we often receive complaints about irresponsible or unfair reviewers. In the first version, the review system was featured by ‘Responsibility + Contribution'. Later, Chinese Vice Premier Liu He pointed out the importance of credit. Indeed, it is hard to imagine a reviewer who does not care about his or her credit will take responsibility or make appropriate contributions.

With credit, the logic of the RCC system became rather clear: credit is the driving force; a credit-cherishing reviewer will evaluate the applications in a responsible way and make concrete contributions to the proposed research work. The reviewer's contributions will then be recorded, which further add to his or her credit. In this way, reviewers build up their own reputation and RCC score day by day and bit by bit. NSFC will give priorities and appreciation to the high-valued reviewers in evaluation. In this way, we hope to make RCC the common value and pursuit of Chinese reviewers.

Meanwhile, the RCC value issue is very complicated, which means we should deal with the RCC data with great caution. The RCC score should be used exclusively in the funding evaluation system and open to reviewers themselves only. It should not be open to the public. In this regard, we need a new Artificial Intelligence (AI)-Assisted Review Management System, as well as a set of strict management rules to guarantee its implementation.

Credit is the driving force; a credit-cherishing reviewer will evaluate the applications in a responsible way and make concrete contributions to the proposed research work.

—Jinghai Li

**NSR:** What will the AI-assisted system be like? Are there any precedents in other countries?

**Li:** There have been prototypes of AI systems, but a complete AI system for funding has not yet been developed. For example, some academic journals are using information technology (IT) systems to match reviewers with the keywords of a manuscript and record the review data of reviewers.

What we would like to do is more advanced, comprehensive and strict. The future system will be able to analyse the research projects and publications of potential reviewers and match them with the proposals through semantic comparison. After identification of reviewers with the most appropriate expertise, the system will further check the credit information and select the high-credit reviewers.

The selection of reviewers is essential in the evaluation of research applications. It is also the common concern of funding agencies in various countries. At the moment the applicants are suspicious of the selection of the IT system, but selection by staff members is often complained as unfair. We hope to gradually solve this problem with the state-of-the-art AI technology. In this respect, we welcome the wisdom of the whole scientific community.

## OPTIMIZE LAYOUT OF RESEARCH AREAS

**NSR:** How would NSFC optimize the layout of research areas?

**Li:** We have proposed two principles for the layout of research areas: first, the new layout should be in accordance with the inherent logic of the knowledge system, and second, it should be in favor of the integration of knowledge and application.

One major problem of the current layout is the rigid separation of research areas. In some cases, similar research projects are classified into different research areas. In consequence, researchers of different research areas, although they are dealing with similar problems, seldom communicate with each other and may even use different terminologies. In fact, many research disciplines are closely interconnected. The layout of research areas is the basis for NSFC to classify applications and select reviewers. Due to historical reasons and limitations of our understanding of the knowledge system in the past, the layout of research areas in use has many defects. This is certainly not helpful for fair and efficient application and evaluation, and also hinders transdisciplinary research.

The knowledge system has its inherent logic and structure. We will re-study this system and build a multi-level layout based on the knowledge nodes, each of which represents a knowledge domain.

In addition, we hope the reform will help to break the barriers between basic and applied research. Every application involves knowledge of different levels. We will take this point into consideration and try to integrate the knowledge system and application into our new layout, thus encouraging researchers to identify and solve the basic scientific problems behind applications.

The optimization of the layout of research areas is a systematic issue. It will have a long-term and wide-ranging impact on the development of China's science and technology, particularly, for transdisciplinarity. We hope to have the understanding, support and help of the scientific community on this issue, and collective wisdom is also needed in this respect.

## ROADMAP OF THE REFORM

**NSR:** What is the roadmap of the reform?

**Li:** If everything goes well, the reform may take 5 years to accomplish. We have drafted a 5–10 years' roadmap, taking the potential difficulties into consideration. There must be difficulties in such a systematic reform of the natural science funding system. But we believe that we will win the support of scientists as long as our reform conforms to the law of scientific development and the measures taken are logically sound. The reform cannot be deemed successful without the support of the scientific community.

In 2019, we will launch the pilot reform in the General Program and the Key Program of the National Natural Science Fund. For the Key Program, the reform will be applied to all the eight scientific departments. For the General Program, the reform will be applied to all disciplines of the Department of Chemical Sciences and one or two disciplines of each of the other departments. If the pilot reform goes well, we will extend the reform to all disciplines and more programs in 2020. [NSFC has altogether eight departments: Department of Mathematical and Physical Sciences, Department of Chemical Sciences, Department of Life Sciences, Department of Earth Sciences, Department of Material Sciences and Engineering, Department of Information Sciences, Department of Management Sciences and Department of Health Sciences.]

The establishment of databases and information systems takes more time and may not be accomplished in 2019. But we are determined to accomplish the basic goals of this reform within 5 years. The most difficult part lies in the optimization of the layout of disciplines, which will take a bit more time.

The reform cannot be deemed successful without the support of the scientific community.

—Jinghai Li

## FUTURE OF THE TALENT PROGRAMS

**NSR:** In June 2018, NSFC released a statement that the National Science Fund for DYS is not a ‘hat' or an honor. But, in spite of this statement, many institutes and researchers do consider it as an honor. What are NSFC's plans for these talent programs?

**Li:** DYS was set up to support the talented young researchers. It is a major task for every funding agency to offer timely support to young talents. DYS has discovered and supported many excellent young researchers ever since it was established more than two decades ago.

However, we must be aware that DYS has its problems. Its honor attribute is overemphasized. Some applicants care more about the academic honor than the funding itself. To pursue the honor, some even take advantage of personal connections during the evaluation process. NSFC is certainly aware of this situation and strives to solve these problems.

After careful consideration, we think it rational to reorganize the talent programs in coordination with other programs into a supporting array. If a young researcher has a good idea, NSFC can support him or her by a Young Scientists (YS) project. If the young researcher obtains excellent outcomes, NSFC can support him or her with an Excellent Young Scientists (EYS) grant or General Program for further research. And if the researcher continues to work well, NSFC can give him or her stronger support through a DYS grant or Key Program. Our expectation is that through the continuous support of this array, an original idea can be developed into a significant research area of international recognition. After that, we can offer further support to this research area, through bigger programs such as Major Program, Creative Research Group Program, Major Research Plan, and Basic Science Center Program, until the accomplishment of world-class research results.

However, the funding he or she received at these stages should simply be considered as support to the research project. It is part of the track record instead of a personal honor or an academic ‘hat' for him or her.

With the development of S&T and the expansion of our scientific community, we are also planning to appropriately adjust the number of grants and the funding amount of DYS.

**NSR:** The current NSFC-funding application is organized by host institutions. The competition among host institutions may somewhat foster the unhealthy atmospheres. Will NSFC consider opening up more application and recommending channels?

**Li:** Yes, we plan to open up new channels of application and are now pondering over the details. For example, maybe the high-credit experts in our system can directly recommend research projects for evaluation. It may provide funding opportunities for non-consensus research. Besides, we hope that host institutions could implement quality control over the applications their researchers submit instead of overemphasizing on the quantity of the applications.

## EXTERNAL CHALLENGES AND BEYOND

**NSR:** Recently, the Tencent Foundation and a number of Chinese scientists set up the Scientific Exploration Award for young scientists. How will the non-governmental funding and awards impact NSFC?

**Li:** Non-governmental funding is part of the multi-source investment system and may have positive effects, which are what we would encourage. On the one hand, it helps to bring down the fervor for DYS and other talent programs. Additionally, it may offer some inspirations to our reform. The increase of non-governmental awards may also play a positive role through changing the atmosphere of over-pursuit of official awards.

**NSR:** Finally, in general, what is the unique role that NSFC could play in China's scientific research?

**Li:** NSFC is China's major funding agency for basic research. We have been following the nature of basic research and holding peer review as the cornerstone in order to support and encourage scientists' free exploration. Therefore, NSFC plays a significant supporting and guiding role for the long-term healthy development of China's disciplinary construction, talent training and scientific research.

Apart from the above-mentioned reform, we are also considering how to change the situation where China lacks original major breakthroughs, and set up a mechanism that could nurture major accomplishments for the next 20–30 years.

Moreover, NSFC should play a guiding role in shaping the scientific spirit, culture and values in China. It has been only 40 years since we started the rapid development of S&T in China. We are still lagging behind the Western countries in terms of scientific spirit, culture and values. By identifying funding categories, improving the evaluation mechanisms and optimizing the layout of research areas, NSFC will be capable of fulfilling our duties to play a guiding role, help the scientific community to build a better scientific culture, promote scientific ethics and integrity, and encourage more researchers to dedicate themselves to major research work that requires long-term hard work. In my opinion, this is also a unique role that NSFC could play.

